# Neem Oil to Reduce Zeatin Use and Optimize the Rooting Phase in *Olea europaea* L. Micropropagation

**DOI:** 10.3390/plants12030576

**Published:** 2023-01-28

**Authors:** Luca Regni, Simona Lucia Facchin, Daniel Fernandes da Silva, Michele De Cesaris, Franco Famiani, Primo Proietti, Maurizio Micheli

**Affiliations:** 1 Department of Agricultural, Food and Environmental Sciences, University of Perugia, Borgo XX Giugno, 06121 Perugia, Italy; 2 Campus Marechal Cândido Rondon, Universidade Estadual do Oeste do Paraná, Rua Pernambuco 1777, Cascavel 85819-110, Brazil

**Keywords:** olive, in vitro culture, complex mixture, cytokinins, substrates composition

## Abstract

Micropropagation is an in vitro propagation technique, established in the nursery field sector for numerous species, which offers several advantages compared to traditional agamic propagation techniques. In the case of the olive tree, however, despite the advances made through research, it is still little used, due to the recalcitrance to in vitro proliferation and/or rooting of many olive cultivars and the high cost of zeatin, the only cytokinin that makes it possible to achieve a satisfactory proliferation rate in this species. In this context, numerous attempts have been made to identify alternative cytokinin compounds able to improve the proliferation rate of olive tree explants and thus reduce the unitary production cost. In particular, there is a growing interest in the use of natural substances (called in some cases “complex mixtures”), which, when added to the in vitro cultivation substrates, seem to be able to improve proliferation rates. In the present study, neem oil was added to the propagation substrates (partially/totally replacing zeatin) and in the rooting phase for the olive cultivar Moraiolo. In particular, in the proliferation phase, the effect of neem oil (0.1 mL L^−1^) in substrates containing different zeatin concentrations (0, 1, 2, and 4 mg L^−1^) was evaluated. For the rooting phase, agarized substrate and soil were used with shoots derived from a standard proliferation substrate (4 mg L^−1^ zeatin) and from the substrate that gave the best results in the proliferation phase (2 mg L^−1^ zeatin and 0.1 mL L^−1^ neem oil). In the proliferation phase, the addition of neem oil in the substrates with low zeatin concentration (1 and 2 mg L^−1^) induced an increase in the number of adventitious shoots and shoots length. On the contrary, the addition of neem oil in the rooting substrates did not positively influence the rooting phase, but positive results especially in terms of root number and length were observed in explants derived from a neem oil-enriched proliferation substrate compared to the control substrate. Therefore, the present study demonstrated for the first time the positive role of neem oil in the proliferation of olive in vitro with low zeatin concentrations.

## 1. Introduction

Olive (*Olea europaea* L.) is an evergreen medium size tree and probably originated from the eastern Mediterranean region of the Middle East (southeastern Anatolian region and south Asia Minor) [1]. The olive tree is one of the most widespread agricultural tree species not only in the Mediterranean basin but recently its cultivation has expanded worldwide also into some nontraditional areas (South Africa, New Zealand, Australia, Chile, etc.) [2]. The world’s harvested area is about 10 million hectares for a total of 23 million tons of olives and 3 million tons of olive oil [3]. In recent years, the spread of *Xylella fastidiosa* infection forcing farmers to plant new olive groves has significantly increased the demand for high genetic and phytosanitary quality olive plantlets [4]. In this context, micropropagation allows the rapid production of quality plants using explants taken from screen houses in conservation centers. Moreover, the production cycle can be carried out in a protected environment, and this allows a high sanitary quality of the plant material produced.

Usually, olive trees are propagated vegetatively by rooting leafy stems or softwood cuttings, by grafting pieces of the stem (scions) onto seedlings or clonal rootstocks or suckers [1]. In particular, the most common method for olive propagation is rooting of leafy stem cuttings under a mist system [5,6] which, however, presents some limitations. Indeed, rooting ability varies depending on the season, cultivars, and availability of healthy viable material, and consequently, the rooting of leafy stem cuttings in some cases is inefficient. To overcome the difficulties connected to the conventional propagation methods, micropropagation was proposed also for olive propagation [7,8].

Micropropagation is used worldwide as an important tool for the large-scale production of clonal plantlets since it has several advantages compared to conventional agamic propagation systems. The main advantages are the high genetic and sanitary quality of the propagated material and the possibility to produce a large number of plants in a small space and in a short time, and this facilitates the plant material exchanges between nurseries [9]. Currently, micropropagation is used for the multiplication of rootstocks and cultivars in several fruit species, such as pear, kiwi, banana, pineapple, and others [3]. Nowadays, the main challenges for micropropagation are represented by cost reduction: for example, using LED systems, efficiency enhancement, ex vitro rooting, use of plastic-free or biodegradable containers, and combining micropropagation with other systems/propagation techniques that can extend its application by replacing the conventional propagation techniques and technologies [9,10,11,12]. However, in the case of olive trees, only a very small percentage of plants is obtained through micropropagation. Indeed, there is difficulty in standardizing the procedures, as the final result seems to be genotype-dependent since olive trees show nutrient and hormonal requirements that vary according to the cultivar [13]. Another limiting factor consists in the ineffectiveness of other regenerative processes (embryogenesis), potentially more productive than multiplication by axillary shoots, but still not safe from the point of view of genetic certification. Regarding the field behavior of micropropagated plants compared to those obtained through traditional techniques, more studies about the possible rejuvenation phenomenon manifested by vitro-derived plants of some cultivars are necessary [8]. Another major problem hindering the spread of micropropagation for olive plants that still makes this technique uneconomic compared to others is the high cost of zeatin, the most effective phytoregulator to obtain satisfactory levels of olive multiplication [13,14,15].

For this reason, particular attention has been paid to the possibility of reducing the use of zeatin. In particular, TIS Plantform™ was tested for olive culture (Canino cultivar) and produced an optimal number of shoots at reduced levels of zeatin [16]. Indeed, a zeatin concentration of 5 µM produces similar results to those obtained with 10 µM in a semi-solid medium. Similar results were obtained by [17] using RITA^®^ system for olive shoots proliferation. Moreover, attempts have been made to identify alternative cytokinins. Meta-Topolin stimulate cytodieresis, providing appreciable results in controlling hyperhydration and delaying senescence, phenomena that can be encountered during olive tree proliferation [18]. Dikegulac at low concentrations and in combination with zeatin promotes the formation of lateral shoots, improving the ability to reduce apical dominance [14]. Moreover, the simultaneous application of dikegulac and white light allowed to overcome apical dominance and to obtain a suitable rate of micropropagation in olive cv Arbequina [19]. BA (6-benzyladenine) was comparable to zeatin for shoot regeneration from nodal explants of the Olive “Gemlik” [1]. The use of a combination of BA μΜ dikegulac enhanced shoot proliferation of olive tree cv “Chondrolia Chalkidikis” and the production of new shoots of good quality, diminishing vitrification symptoms [20]. Additionally, BAP (6-benzylaminopurine) and 2iP (dimethyl-allyl-aminopurine) were tested but, on some cultivars, produced results inferior to zeatin at the end of proliferation [21] or, as in the case of BAP, provided the best results in combination with zeatin [22]. On the contrary, [23] proved that BAP is a good hormonal supplement for Leccino, Gemlik, Moraiolo, and Arbosana cultivars if combined with pre-cooling treatments. A combination of coconut water, 2ip, and kinetin under different light intensity was successfully tested for olive regeneration through direct organogenesis [24].

In addition to hormonal substances, organic nitrogens, organic acids, and natural extracts with species-specific effects, such as water and/or coconut milk, casein hydrosylate, malt extracts, tomato extracts, potato extracts, banana homogenate, yeast extract [25] algal compounds [26,27], experimented in other species can be used also for olive to solve the problem of the high cost of zeatin. Among the substances of natural origin that can potentially be added to growth media for olive in vitro propagation, there is neem oil also. Indeed, a previous article, [10] demonstrated that the addition of neem oil to an Olive Medium (OM) containing zeatin (4 mg L^−1^) increased the shoot length, the number of nodes, and the fresh and dry weight of shoots of olive cultivar Moraiolo.

Neem oil is usually prepared from the seed kernels of *Azadirachta indica* A. Juss, belonging to the *Meliaceae* family that originates from the Indian subcontinent [28] and is well known worldwide as an important source of phytochemicals for human health and in agriculture [29]. In particular, in the agricultural sector, neem oil can be used as a soil conditioner, fumigant, and pesticide [29,30]. In particular, the addition of neem oil to soil led to a significant increase in the population of nitrogen fixers and better growth and vigor of the plants [31]. Additionally, neem seed cake (residue of neem seeds after oil extraction) can be successfully used in agriculture. Indeed, it is reported in various studies that neem cake in combination with urea can reduce the fertilizer nitrogen requirement [30]. However, according to a part of the abovementioned paper [32], the knowledge about the effects of neem oil in in vitro plant culture is still scarce. In this context, the aim of the present work was (i) to study the effect of adding neem oil to proliferation substrates, with reduced zeatin concentrations for olive explants, and (ii) to optimize the rooting phase by using neem oil in different types of substrates.

## 2. Results

### 2.1. Proliferation Phase Experiment

In the Z0 substrate, the viability of explants decreased to zero during the second subculture, while in the Z0 n.o. substrate the viability of explants decreased to zero during the third subculture. Therefore, explants’ growth data for the substrates Z0 and Z0 n.o. were not considered. In all other substrates and during the three subcultures, a satisfactory level of explant viability was recorded. In particular, in the Z4 substrate, a viability value equal to 100% was recorded and this value was not statistically different values from that registered for the Z4 n.o. (98.9%) and Z2 n.o. (98.9%) substrates. On the other hand, the viability values observed in Z1 and Z1 n.o. (92.1 and 92.8%, respectively) were statistically lower.

The number of shoots in the Z2 and Z1 substrates was lower than that observed in the Z4 substrate, where, generally, the development of both axillary buds present on the explant’s node was observed.

**Figure 1:**
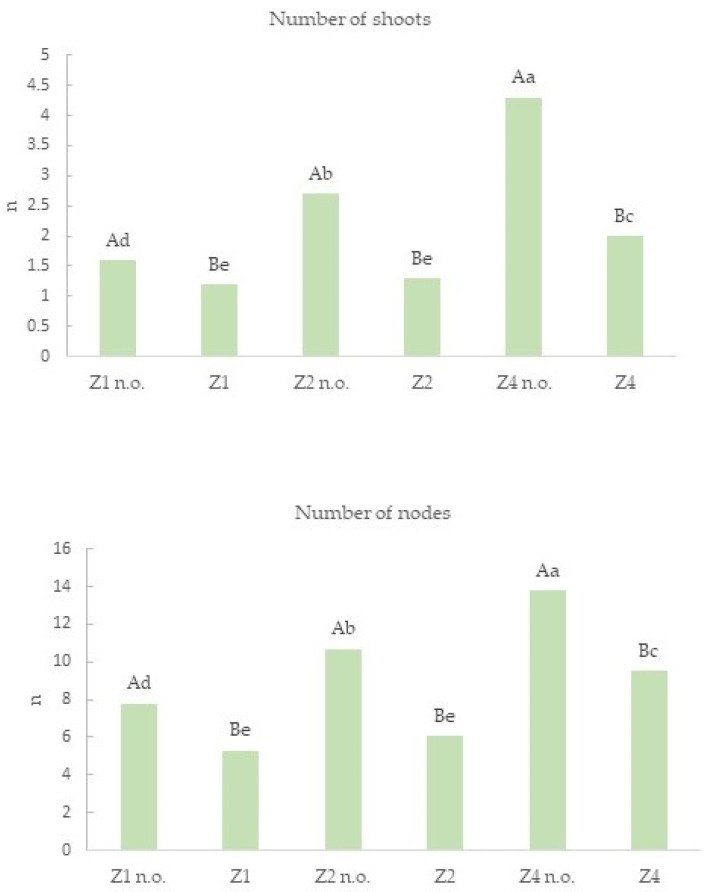
Number (*n*) of shoots and nodes produced by each initial explant. The values followed by a different capital letter are statistically different according to Duncan’s test (*p* ≤ 0.05) for each treatment with the same zeatin concentration; the values followed by a different small letter are statistically different in the comparison of all treatments.

**Figure 2:**
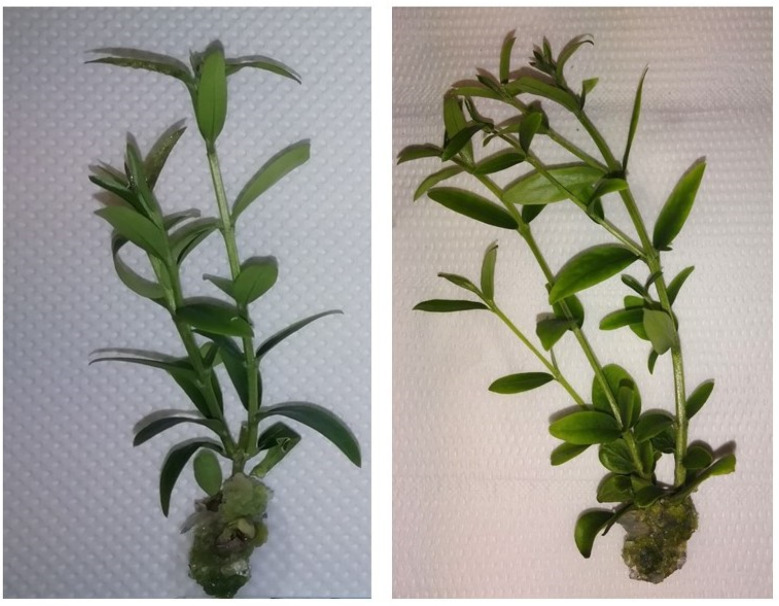
Olive explants grown in Z4 n.o. (zeatin 4 mg L^−1^, neem oil 0.1 mL L^−1^) (**right**) and Z4 (zeatin 4 mg L^−1^, neem oil 0 mL L^−1^) (**left**) substrates.

**Figure 3:**
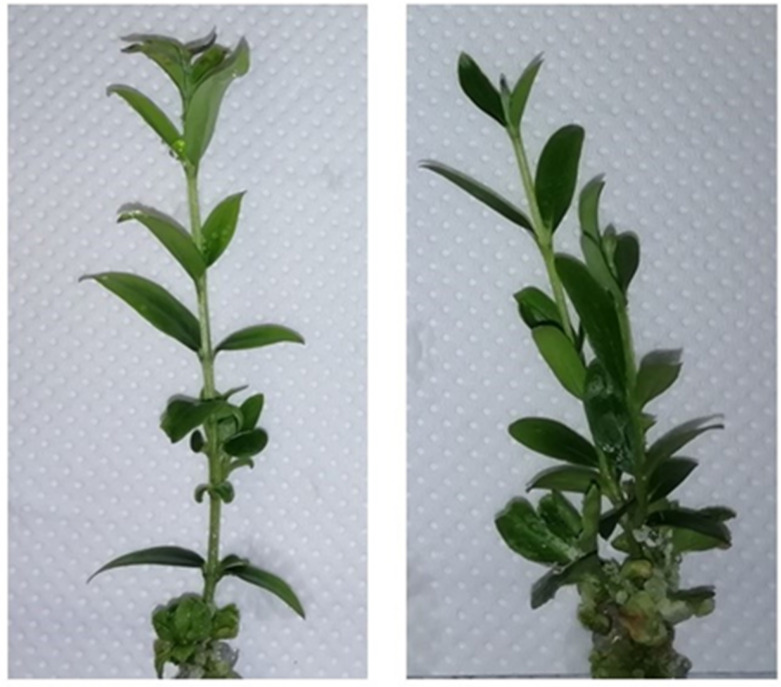
Olive explants grown in Z2 n.o. (zeatin 2 mg L^−1^, neem oil 0.1 mL L^−1^) (**right**) and Z2 (zeatin 2 mg L^−1^, neem oil 0 mL L^−1^) (**left**) substrates.

The addition of neem oil caused an increase in the number of shoots produced; indeed, in the Z4 n.o. and Z2 n.o. substrates, the values of more than two shoots were recorded (Figure 1, Figure 2 and Figure 3). Therefore, the addition of neem oil stimulated the development of the adventitious buds present on the two main shoots. The neem oil also increased the number of nodes in all the substrates and higher values were observed for Z4 n.o. and Z2 n.o (Figure 1).

At the lowest concentration of zeatin (1 mg L^−1^), the addition of n. o. strongly increased the shoot elongation (from 27.3 to 47.7 mm) (Table 1). In the Z2 and Z2 n.o. substrates, the n.o. does not increase the shoot length, while in the Z4 substrate the addition of neem oil leads to a decrease in the shoot length (Table 1).

The addition of n.o. increased the number of leaves produced by each initial explant in all the substrates. In particular, the highest number of leaves was recorded in Z4 n.o. followed by Z2 n.o. and Z1 n.o (Table 1).

**Figure 4:**
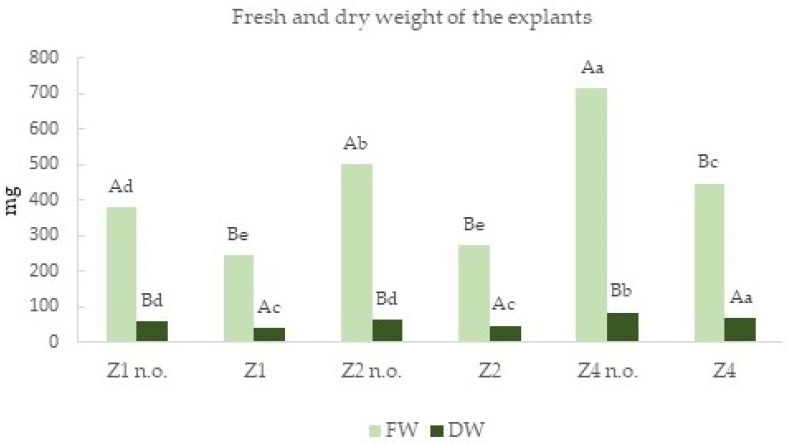
Fresh weight (FW) and dry weight (DW) per explant. The values followed by a different capital letter are statistically different according to Duncan’s test (*p* ≤ 0.05) for each treatment with the same zeatin concentration; the values followed by a different small letter are statistically different in the comparison of all treatments.

The data on the fresh and dry weights (Figure 4) per explant confirm the positive effect of neem oil on olive tree growth in vitro. This occurs with all zeatin concentrations, although it is most evident when zeatin is at the standard level (4 mg L^−1^).

Therefore, the addition of neem oil induced the development of adventitious shoots, a greater: shoots’ length, leaves number, and plant fresh and dry weights.

### 2.2. Rooting Phase Experiment

This experiment aimed to investigate whether the addition of neem oil could have a residual effect (for the shoots derived from the Z2 with n.o. (Z2 n.o.) substrate) or a direct effect on the rhizogenic capacity of the shoots when added to the rooting substrate. For shoots from the Z2 n.o. substrate, the highest rooting percentage was recorded in the potting substrate (P) without the addition of n.o., whereas for shoots from the Z4 substrate, the highest rooting percentage was recorded in the black agarized medium without n.o. (b) and with n.o. (b n.o.) and in the potting substrate (p). In general, the lowest rooting values were recorded for shoots from Z4 in the white (w) and white n.o. (w n.o) agarized substrates (Table 2). The addition of n.o. to the substrates had no positive effect on the rooting percentage, which was equal to or lower than the substrates without n.o. in the different substrates. The highest value for the number of roots produced was recorded for shoots from the Z2 n.o. substrate in the P substrate (Table 2).

The potting substrate with shoots from Z2 n.o. provided the highest percentage of rooting and the highest number of roots produced (Table 2). In general, with regard to the average root production per shoot, when comparing the different rhizogenic treatments, the addition of n.o. to the rooting substrate, in most cases, seems to lead to a decrease in the number of roots produced. This would suggest that it is not necessary to treat shoots with n.o. to induce rooting. On the contrary, the use of shoots from the Z2 n.o. substrate almost produced better results, given the same rhizogenic treatment and substrate used in terms of rooting percentage and roots’ length, compared to those obtained from shoots proliferated in the Z4 substrate. On the contrary, the longest root length was found in the W treatment (OM /2 white agarized substrate), in the absence of n.o., suggesting that the direct exposure of n.o. negatively influences the roots’ length.

## 3. Discussion

Olive micropropagation is generally limited due to the strong apical dominance limiting secondary axillary shoot formation [32,33]. Uninodal explants usually produce one shoot; consequently, olive shoot multiplication is performed by segmentation of elongated shoots at each subculture. The addition of n.o. at a concentration of 0.1 mL L^−1^ increased the number of shoots produced, even at the zeatin concentrations of 2 mg L^−1^. The obtained results suggest that the addition of n.o. also stimulated the development of adventitious buds present on the two main shoots, since an average number of shoots of more than two was observed. This could be interpreted as the ability of this substance to promote the growth of olive explants in vitro [32]. Indeed, [32] reported that the addition of neem oil at a low concentration (0.1 mL·L^−1^) to the nutritive medium (zeatin concentration 4 mg·L^−1^) was able to improve the shoot length, the multiplication rate, and fresh and dry weights of the proliferated explants. Neem oil contains at least 100 biologically active compounds: the major constituents are triterpenes known as limonoids, the most important being azadirachtin, while other components include meliantriol, nimbin, nimbidin, nimbinin, nimbolides, fatty acids, and salannin [28]. Thanks to its complex chemical composition, neem oil would appear to act in synergy with the nutrient components of the growth medium, improving its trophic function and simulating the effects of gibberellins and cytokinins such as a higher shoot length and the development of secondary adventitious shoots. The breaking of apical dominance was observed in the Manzanillo cultivar using mannitol [34], ZnO nanoparticles in the Moraiolo cultivar using [35], and dikegulac and white light for the cultivar Arbequina [19].

The results on the viability of the explants confirm that zeatin is an indispensable element for olive tree micropropagation, although there is the possibility that it can be used at a lower concentration than the standard (4 mg L^−1^). Indeed, the present study shows that, at 2 mg L^−1^, zeatin in substrates with neem oil results in an explants’ viability similar to the control. In general, the use of zeatin in the proliferation phase promotes a higher number of shoots and nodes, a higher shoot length and number of leaves and, as a consequence, a higher proliferated shoot fresh and dry weights. In another study, coconut water in combination with BAP successfully replaced commercial zeatin in Galega vulgar cultivar [36]. Coconut water is known as a natural substance with high zeatin content and induces plant cells to divide and grow rapidly; and for these reasons, it was successfully used also for passion fruit [37] and orchid [38] micropropagation. However, the coconut water without BAP was not sufficient to promote satisfactory multiplication, and so it seems that the association of different cytokinins is the best way to improve the multiplication rates [36]. Additionally, in our experiment, neem oil without the cytokinin zeatin did not give satisfactory results. Indeed, the viability of explants decreased to zero in a few subcultures in zeatin-free media also with neem oil addition. Regarding the use of BAP, the results are contrasting. Indeed, according to some authors [22], the best results were obtained only when BAP is applied together with zeatin, while other authors found that BAP in combination with pre-cooling treatments can be used instead of zeatin for several olive cultivars [23].

The addition of n.o. to the different substrates had no positive effect on the rooting parameters. However, the use of shoots deriving from a reduced zeatin concentration (2 mg L^−1^) produced better results in terms of root development, given the same rhizogenic treatment and substrate used, compared to those obtained from shoots proliferated in the control substrate (zeatin concentration 4 mg L^−1^). Regarding the optimization of the rooting phase, the addition of putrescine (1,4-diaminobutane) in combination with IBA, has been the subject of interest for its root-promoting effect [21]. In our work, n.o. does not appear to have a positive effect on rhizogenesis when added directly to the substrate, whereas positive effects were found in shoots from proliferation substrates containing n.o. Therefore, the improvement in rooting parameters is probably related to the fact that stronger shoots produced in proliferation substrates containing neem oil are more capable of producing a good root system.

## 4. Materials and Methods

### 4.1. Proliferation Phase Experiment

The experiment was carried out in 2020 in the Laboratory of Micropropagation and In Vitro Biotechnology of the Department of Agricultural, Food and Environmental Sciences—University of Perugia, Italy. The experiment conducted on the proliferation phase involved the comparison of eight olive media (OM) Duchefa [33] (Table 3) substrates that differed from each other in the concentration of zeatin (Z) and the presence/absence of neem oil (n.o.), as shown in Table 4. In particular, the numbers after the letter Z (0, 1, 2 and 4) indicate the zeatin concentration (mg L^−1^), and in the substrate characterized by the presence of neem oil “n.o.” was added in the acronym. The neem oil concentration was chosen on the basis of a precedent experiment [32].

All substrates were enriched with sucrose (30 g L^−1^), agar (7 g L^−1^) and buffered to a pH of 5.5. Neem oil concentration was established based on a previous study [32]. In particular, a neem oil provided by “Neem Italia company” composed by neem seed oil (from organic agriculture) and Polysorbate 85 was used. Each vessel (500 mL volume) contained 100 mL of the OM substrate. The substrates and vessels were autoclaved at 115 °C for 20 min before being used in aseptic conditions under a horizontal laminar flow cabinet. Olive explants of the Moraiolo olive cultivar obtain from a 5-year-old donor tree were used. The initial explants were represented by about 10 mm long portions with a single node. The shoots’ apical portions were not used since the apical bud is more vigorous due to the apical dominance. The cultures were then placed in a growth chamber at a 22 ± 2 °C temperature and a 16 h photoperiod with a light intensity of 40 µE m^−2^ s^−1^. For each treatment, six vessels (replicates) for each one of the three subsequent subcultures were used. At the end of each subculture (45 days), this was allowed to carry out the destructive measurements on the proliferated shoots of three pots and to use the proliferated shoots of the remaining vessels as initial explants for the subsequent subcultures.

The following parameters were measured at the end of each subculture:

- viability (%): incidence of green and viable explants;
- shoots (n): average number of shoots developed from each initial explant;
- shoot length (mm): average length of developed shoots;
- nodes (n): average number of nodes developed by each initial explant, reusable for further proliferation subculture (multiplication rate);
- leaves (n): average number of leaves;
- callus (%): incidence of explants that produced basal callus;
- fresh weight (mg): average fresh weight per explant of developed vegetative organs (leaves, stems, buds) and callus;
- dry weight (mg): average dry weight per explant of the vegetative organs and callus, obtained by keeping the plant material in an oven for three days at 105 °C.

### 4.2. Rooting Phase Experiment

For the rooting experiment, shoots from Z4 and Z2 n.o. proliferation substrates were used. The latter was chosen because, among the substrate with reduced zeatin concentration used in the proliferation experiment was the one that gave the best results. The details of the experimental framework for the rooting experiment are shown in Table 5. In particular, “w” and “b” refer to the color of the agarized substrate (white and black, respectively), while “P” was used for the potting substrate. The initial capital letter in the acronym was used for the explants derived from a proliferation substrate (OM) with 2 mg L^−1^ of zeatin and neem oil (0.1 mL L^−1^). On the contrary, the initial lowercase letter in the acronym was used for the explants derived from a proliferation substrate (OM) with 4 mg L^−1^ of zeatin. Moreover, in the rooting substrates characterized by the presence of neem oil, n.o. was added in the acronym.

In particular, OM/2 white is a rooting medium consisting of the OM substrate at half concentration of all components (including sucrose) with agar as gelling agent, while OM/2 black is a rooting medium with the same characteristics of OM/2 white but with Brilliant Black^®^ (150 mg L^−1^) added. The potting substrate, instead, consists of a mixture of “Solevivo cactacee (AL.FE srl)” + sand (3:1, *v*:*v*) and moistened with liquid OM/2 (20% in volume) (Table 6). Regarding the concentration of IBA used, the OM/2 substrates were enriched with 2 mg L^−1^ while in the potting substrate the shoots, before being placed on the substrate, were subjected to an inductive rhizogenic treatment, keeping their base immersed in a solution of IBA (5 mg L^−1^) and sucrose (15 g L^−1^) in the dark, for 3 days, at room temperature.

All substrates were autoclaved for 20 min at 115 °C. Three pots with ten uninodal explants for each treatment were used and placed in the same growth chamber described for the proliferation experiment. After 45 days, the following parameters were monitored:

- viability (%): incidence of green and viable explants;
- rooting (%): incidence of explants that develop roots;
- roots (n): average number of roots developed by each explant;
- roots length (mm): average length of developed roots;
- callus (%): incidence of explants that produced basal callus;

### 4.3. Statistical Analysis

The trials were organized according to a completely randomized design and collected data were subjected to analysis of variance (ANOVA). Significant differences were assayed by Duncan’s test (*p* = 0.05).

## 5. Conclusions

From the present study, it can be concluded that the substrate containing zeatin at 2 mg L^−1^ with neem oil (0.1 mL L^−1^) can be considered the best performing of those with a reduced zeatin concentration. This is particularly interesting due to the high cost that the use of zeatin at the higher concentration (4 mg L^−1^) implies. Indeed, the cost of the amount of neem oil needed to replace zeatin is about one thousandth of the cost of the latter. The increased shoot length obtained by adding neem oil to the substrates is a quality parameter of great importance in commercial micropropagation. Indeed, obtaining well-developed shoots at the end of the proliferation phase provides material that is certainly more suitable for the rooting phase. On the contrary, small explants (which would be excellent for new proliferation cycles) may provide worst performances during rhizogenesis, or once rooted provide smaller plantlets, with difficulties in the acclimatization phase. The direct neem oil use in the rooting phase does not seem to provide any beneficial effects, while positive effects in terms of rooting percentage and root length were observed in shoots deriving from the neem oil-enriched proliferation substrate.

## Figures and Tables

**Table 1:** Average values for shoot length and number of leaves by each initial explant.

Treatment	Shoot Length (mm)	Leaves (n)
Z1 n.o.	37.7 Aa	17.6 Ac
Z1	27.3 Bc	11.6 Be
Z2 n.o.	31.0 Ac	21.7 Ab
Z2	31.5 Ab	13.7 Bd
Z4 n.o.	24.4 Bd	27.8 Aa
Z4	39.7 Aa	19.7 Bb

The values of each column followed by a different capital letter are statistically different according to Duncan’s test (*p* ≤ 0.05) for each treatment with the same zeatin concentration; the values followed by a different small letter are statistically different in the comparison of all treatments.

**Table 2:** Parameters related to root development.

Treatment	Provenience Substrate of Shoots	Rooting (%)	Roots Number (n)	Roots Length (mm)
W n.o.	Z2 n.o.	86.6 c	2.5 c	32.1 b
W	Z2 n.o.	83.3 d	2.1 e	34.5 a
B n.o.	Z2 n.o.	85.0 e	2.6 c	13.5 f
B	Z2 n.o.	96.7 a	3.0 b	20.6 d
P n.o.	Z2 n.o.	80.0 e	1.9 f	12.1 g
P	Z2 n.o.	100.0 a	3.8 a	14.6 e
w n.o.	Z4	40.0 f	1.7 g	19.3 d
w	Z4	40.0 f	1.3 h	14.5 e
b n.o.	Z4	96.7 a	2.3 d	20.5 d
b	Z4	100.0 a	3.0 b	19.3 d
p n.o.	Z4	95.0 b	2.0 e	15.7 e
p	Z4	100.0 a	2.3 d	22.6 c

The values of each column followed by a different letter are statistically different according to Duncan’s test (*p* ≤ 0.05).

**Table 3:** Chemical composition of Ruggini Olive Medium (Duchefa).

**Macroelements**	**mg L^−1^**	**mM**
CaCl_2_	332.16	2.99
Ca(NO_3_)_2_	416.92	2.54
KCl	500.00	6.71
KH_2_PO_4_	340.00	2.50
KNO_3_	1100.00	10.88
MgSO_4_	732.60	6.09
NH_4_NO_3_	412.00	5.15
**Microelements**	**mg L^−1^**	**μM**
CoCl_2_.6H_2_O	0.025	0.11
CuSO_4_.5H_2_O	0.25	1.00
FeNaEDTA	36.70	100.00
H_3_BO_3_	12.40	200.55
KI	0.83	5.00
MnSO_4_.2H_2_O	16.90	100.00
NaMoO_4_.2H_2_O	0.25	1.03
ZnSO_4_.7H_2_O	14.30	49.75
**Vitamins**	**mg L^−1^**	**μM**
Biotin	0.05	0.20
Folic acid	0.50	1.13
Glycine	2.00	26.64
myo-Inositol	100.00	554.94
Nicotinic acid	5.00	40.62
Pyridoxine HCl	0.50	2.43
Thiamine HCl	0.50	1.48

**Table 4:** Substrates used for the proliferation phase and their characteristics in terms of zeatin (Z) concentration and neem oil (n.o.) presence.

Treatment	Substrate	Zeatin (Z) Concentration (mg L^−1^)	Neem Oil (n.o.) Concentration (mL L^−1^)
Z0 n.o.	OM	0	0.1
Z0	OM	0	0
Z1 n.o.	OM	1	0.1
Z1	OM	1	0
Z2 n.o.	OM	2	0.1
Z2	OM	2	0
Z4 n.o.	OM	4	0.1
Z4	OM	4	0

**Table 5:** Substrates used for the rooting phase and their characteristics in terms of IBA concentration and neem oil (n.o.) presence.

Treatment	Proliferation Substrate	Rooting Substrate	IBA Concentration (mg L^−1^)	n.o. Concentration (mL L^−1^)
W n.o.	Z2 n.o.	OM/2 white	2	0.1
W	Z2 n.o.	OM/2 white	2	0
B n.o.	Z2 n.o.	OM/2 black	2	0.1
B	Z2 n.o.	OM/2 black	2	0
P n.o.	Z2 n.o.	potting substrate	5	0.1
P	Z2 n.o.	potting substrate	5	0
w n.o.	Z4	OM/2 white	2	0.1
w	Z4	OM/2 white	2	0
b n.o.	Z4	OM/2 black	2	0.1
b	Z4	OM/2 black	2	0
p n.o.	Z4	potting substrate	5	0.1
p	Z4	potting substrate	5	0

**Table 6:** Physico-chemical characteristics of potting substrate used for the rooting experiment.

Physico-Chemical Characteristics	
pH	7.5
Electrical conductivity	0.44 dS m^−1^
Bulk density	949 kg m^−3^
Total porosity	62% (*v:v*)

## Data Availability

The data that support the findings of this study are available from the corresponding author upon reasonable request.
